# Supplementary material for: Racial and ethnic disparities in “stop-and-frisk” experience among young sexual minority men in New York City
Source: PLoS One. 2021 Aug 26;16(8):e0256201. doi: 10.1371/journal.pone.0256201 (PMC8389488; doi:10.1371/journal.pone.0256201)
Supplement: Attachment — Submitted filename: Response to Reviewers.docx [file pone.0256201.s002.docx]

Response to Reviewers’ Comments

**1) Please ensure that your manuscript meets PLOS ONE's style requirements, including those for file naming. The PLOS ONE style templates can be found at**

https://journals.plos.org/plosone/s/file?id=wjVg/PLOSOne_formatting_sample_main_body.pdf and

[https://journals.plos.org/plosone/s/file?id=ba62/PLOSOne_formatting_sample_title_authors_affiliations.pdf](about:blank)

We have addressed all formatting inconsistencies.

**2) Please state whether the data utilized in this study were de-identified/anonymised before access?**

The data were de-identified (removal of participant name, address) with the exception of ZIP code of residence which enabled ZIP code-level analysis.

**3) In statistical methods, please refer to any post-hoc corrections to correct for multiple comparisons during your statistical analyses. If these were not performed please justify the reasons. Please refer to our statistical reporting guidelines for assistance (https://journals.plos.org/plosone/s/submission-guidelines.#loc-statistical-reporting).**

The paper was largely descriptive hence multiple comparisons were not considered. We aimed to describe the race disparities in stop-and-frisk among YSMM. We calculated RRs and 95% confidence intervals to describe differences between Black versus white and Latinx versus white participants on prevalence and rates of stop and frisk experience. We were not conducting multiple tests of significance.

**4) In your statistical analyses, please describe how you accounted for clustering of data by locality. For example, did you use or consider using multilevel models?**

Study participants were recruited from the New York City area. We accounted for geographic differences by mapping stop-and-frisk by geographic location and to compare race differences in levels of stop-and-frisk across different geographic settings defined by neighborhood-level stop-and-frisk rate.

**5)  We note that the grant information you provided in the ‘Funding Information’ and ‘Financial Disclosure’ sections do not match.**

We have addressed this inconsistency.

**When you resubmit, please ensure that you provide the correct grant numbers for the awards you received for your study in the ‘Funding Information’ section.**

We have ensured the correct grant numbers have been indicated: National Institute on Drug Abuse grant ‘Stop-and-Frisk, Arrest, and Incarceration and STI/HIV Risk in Minority MSM’ (Principal Investigator: Maria Khan; R01 DA044037) and the P18 Cohort Study Grant (Principal Investigator: Perry Halkitis; 1R01DA025537 and 2R01DA025537).

**6)  We note that Figure 3 in your submission contain map images which may be copyrighted. All PLOS content is published under the Creative Commons Attribution License (CC BY 4.0), which means that the manuscript, images, and Supporting Information files will be freely available online, and any third party is permitted to access, download, copy, distribute, and use these materials in any way, even commercially, with proper attribution. For these reasons, we cannot publish previously copyrighted maps or satellite images created using proprietary data, such as Google software (Google Maps, Street View, and Earth). For more information, see our copyright guidelines: http://journals.plos.org/plosone/s/licenses-and-copyright.**

Figure 3 was created by the authors using P18 participant data. It is not a copyrighted image.

**Reviewer #1: Overall, I believe this is an important contribution to the literature on health and social disparities among sexual minority men. This is a well written paper and contains compelling findings for public health practice and public safety. The findings further document racial/ethnic inequities in policing and present new and compelling data related to community policing and sexual minority men.**

We appreciate the enthusiasm for this work.

**Reviewer #2: You have provided data analysis for a space otherwise unresearched. Few, if any, authors are performing analysis and discussion on YSMM as it relates to stop and frisk. This manuscript has added an intersectional layer for race, gender, and other sociodemographic factors which makes the research distinct from extant knowledge. The results were congruent with findings from current literature regarding gender and race while complementing the area with data for YSMM. In the text, you explained an outlier that reported 66 stops in one year. More explanation on that person's experiences would be ideal in determining whether he/she/they had extenuating circumstances (such as activism, recidivism, etc) that may offer insight on other intersectional factors, which could possibly open the door for future research.**

We have had internal discussion about how to best address the comment given some of our growing concerns about deductive disclosure of this individual as a result of providing too much information on him. Since the reviewer expressed interest in intersectional stigma we elected to provide some details on the individuals' psychosocial vulnerability and PTSD risk but avoided additional demographic, socioeconomic factors, sex trade involvement etc. We added this information to the results and a note in the discussion to reflect on results.
